# Olive Leaf Extract from Sicilian Cultivar Reduced Lipid Accumulation by Inducing Thermogenic Pathway during Adipogenesis

**DOI:** 10.3389/fphar.2016.00143

**Published:** 2016-05-31

**Authors:** Rosa Palmeri, Julieta I. Monteleone, Giovanni Spagna, Cristina Restuccia, Marco Raffaele, Luca Vanella, Giovanni Li Volti, Ignazio Barbagallo

**Affiliations:** ^1^Department of Agricultural, Food and Environment, University of CataniaCatania, Italy; ^2^Biochemistry Section, Department of Drug Sciences, University of CataniaCatania, Italy; ^3^Department of Biomedical and Biotechnological Sciences, University of CataniaCatania, Italy; ^4^Euro-Mediterranean Institute of Science and TechnologyPalermo, Italy

**Keywords:** olive leaf extract, thermogenesis, adipocyte, lipid metabolism, stem cells differentiation, heme oxygenase

## Abstract

Olive leaves contain a wide variety of phenolic compounds belonging to phenolic acids, phenolic alcohols, flavonoids, and secoiridoids, and include also many other pharmacological active compounds. They could play an important role in human diet and health because of their ability to lower blood pressure, increase coronary arteries blood flow and decrease the risk of cardiovascular diseases. The aim of this study was to investigate the effect of olive leaf extract (OLE) from Sicilian cultivar on adipogenic differentiation of human adipose derived mesenchymal stem cells and its impact on lipid metabolism. We showed that OLE treatment during adipogenic differentiation reduces inflammation, lipid accumulation and induces thermogenesis by activation of uncoupling protein uncoupling protein 1, sirtuin 1, peroxisome proliferator-activated receptor alpha, and coactivator 1 alpha. Furthermore, OLE significantly decreases the expression of molecules involved in adipogenesis and upregulates the expression of mediators involved in thermogenesis and lipid metabolism. Taken together, our results suggest that OLE may promote the brown remodeling of white adipose tissue inducing thermogenesis and improving metabolic homeostasis.

## Introduction

Olive leaves contain a wide variety of phenolic compounds belonging to phenolic acids, phenolic alcohols, flavonoids, and secoiridoids, and include also many other pharmacological active compounds such as oleuropein (OE), hydroxytyrosol (HT), tyrosol, cumaric acid, ferulic acid, caffeic acid, vanillic acid, rutin, verbascoside, luteolin, quercetin, dimethyloleuropein, and ligstroside (Talhaoui et al., 2015). In particular, OE content, the major phenolic compound of olive leaves, varies from 17 to 23% (Le Tutour and Guedon, 1992). These phenolic compounds have a significant pleiotropic effects including antioxidant activity (Somova et al., 2003) as well as antimicrobial activity against *Helicobacter pylori*, *Campylobacter jejuni*, *Staphylococcus aureus* (Sudjana et al., 2009), and *Bacillus cereus*, *Escherichia coli*, and *Salmonella enteritidis* (Lee and Lee, 2010). Furthermore, they play an important role in human diet and health because of their ability to lower blood pressure, increase blood flow in the coronary arteries, and decrease the risk of cardiovascular diseases (Khayyal et al., 2002). Most of the studies attribute biological activities of olive leaf extracts (OLEs) to total phenols or individual phenolic compounds (Al-Azzawie and Alhamdani, 2006) and to flavonoids and their derivatives (Goulas et al., 2010). Benavente-Garcia et al. (2002) demonstrated that olive phenolics show a synergic behavior in their radical scavenging capacity. Recently, it has been demonstrated that OLE attenuates obesity in high-fat diet-fed mice by modulating the expression of molecules involved in adipogenesis and thermogenesis (Shen et al., 2014). In particular, this study demonstrates that OLE exerts beneficial effects against obesity by overexpressing sirtuin 1 (SIRT-1), peroxisome proliferator-activated receptor alpha (PPARα), and peroxisome proliferator-activated receptor gamma (PPARγ), coactivator 1 alpha (Pgc-1α).

Thermogenesis contributes to ameliorate the metabolic rate as well as glucose and lipid metabolism in humans (Sacerdoti et al., 2005; Kajimura and Saito, 2014) and it represents the distinctive feature of adipocytes contained in the brown adipose tissue (BAT). Two types of adipose tissue are known, white (WAT) and brown (BAT) adipose tissue. WAT is widely distributed throughout the body and its function is to store excess energy as fat while BAT, observed only in limited parts of the body such as interscapular, axillary, superior cervical, and perirenal regions, possesses the ability to consume excess energy as heat (thermogenesis). BAT is densely populated with mitochondria containing the inner mitochondrial proton carrier uncoupling protein 1 (UCP1) that uncouples oxidative phosphorylation, allowing the mitochondrial membrane potential to be dissipated as heat (Rousset et al., 2004).

The impact of adipocyte differentiation and the expansion of particular adipose tissue depots, in view of the different functions of these depots, have important physiological and pathophysiological significance. WAT dysfunction is associated with insulin resistance, low grade inflammation, dyslipidemia, and cardiometabolic risk. By contrast, brown fat enhances thermogenesis, lipid oxidation, insulin sensitivity, and glucose tolerance (Diaz et al., 2014; Poher et al., 2015).

Recent findings have demonstrated that following specific stimuli, WAT may switch its own metabolism and phenotype in the ‘brown-like’ adipocytes, also known as beige cells (Barbagallo et al., 2015; Cohen and Spiegelman, 2015). Many genes and pathways that regulate brown and beige adipocyte biology have now been identified, providing a variety of promising therapeutic targets for metabolic disease (Cohen and Spiegelman, 2015).

The aim of this study was to investigate the effect of OLE on adipogenic differentiation of human adipose derived mesenchymal stem cells and to assess the role of the extract on the switching of adipocytes from white to brown phenotype and its impact on thermogenesis.

## Materials and Methods

### Sample Preparation

Samples of olive leaves (*Olea europaea L*.) from four sicilian varieties namely ‘Biancolilla’, ‘Coratina’, ‘Nocellara’, and ‘San Benedettese’ were employed. The leaves were collected at the same time, during pruning period, from Sicilian organic farming.

The extract was obtained in accordance to Lee-Huang et al. (2003) method with some modifications. Briefly, sicilian olive leaves (OLEs), previously dehydrated at 40°C, were extracted by an aqueous solvent, 100 mL of hot water was employed for the extraction of 5 g of leaves. Subsequently, the samples were stored in the dark.

### Total Polyphenols Content

Total polyphenol content of OLEs was evaluated by Folin–Ciocalteu method (Vazquez-Roncero et al., 1973), with some modifications. Briefly, 1.25 mL portion of Folin–Ciocalteu (Fluka Analytical) reagent was mixed with 0.25 mL of the sample; after 3 min, 2.5 mL of a sodium carbonate solution (20%) was added to the mixture and the reaction was kept in the dark for 1 h. The absorbance was spectrophotometrically measured at 725 nm, using Perkin elmer lambda 25 uv-vis spectrometer. Caffeic acid (Fluka) was used as reference standard for calibration curve (0.02–0.9 mg/mL; *y* = 1.1429*x* + 0.0185, where *x* and *y* represent the caffeic acid concentration (mg/mL) and absorbance at 725 nm, respectively; *r*^2^ = 0.9995). Contents of total phenolic compounds in OLE were expressed as caffeic acid equivalents in milligram per gram of dried leaf (Vazquez-Roncero et al., 1973).

### Antioxidant Capacity

Radical scavenging activity was determined on OLE from different cultivar by the DPPH as previously described (Brandwilliams et al., 1995). Briefly, a methanol 1,1-Diphenyl-2-picryl-hydrazyl DPPH• (Sigma–Aldrich, USA) solution (100 μM) was used and 3 mL of solution was mixed with 70 μL of each leaf extract. The samples were incubated for 60 min at room temperature, then the decrease in absorbance at 515 nm (*A*E) was measured spectrophotometrically. DPPH radicals have a maximum absorption at 515 nm, the peak disappears with reduction by an antioxidant compound. A blank sample, containing 70 μL of methanol in the DPPH• solution, was used as reference. The experiment was carried out in triplicate and Trolox equivalent antioxidant capacity (TEAC) value was derived for each OLE. In addition Radical scavenging activity (RSA%) was calculated using the following equation:

RSA% = [(AB−AE)/AB]× 100

*A*B, absorbance of the blank sample, and *A*E, absorbance of the leaf extract. RSA% values were expressed as μmol Trolox equivalents/g of leaf extract.

### Adipose Stem Cells Isolation and Culture

Subcutaneous adipose tissue was obtained from a 23-year-old man with no significant medical history undergoing umbilical hernioplasty. Written consent was obtained. Since this is a non-therapeutic trial, it was carried out with the consent of the subject legally acceptable according our Italian Government (Legge 675/1996 and DL 196/2003, art. 40. Art 32 Codice Italiano di Deontologia Medica). Adipose tissue was minced with scissors and scalpels into less than 3-mm pieces and isolation of ASCs proceeded as previously described (Salomone et al., 2013). Briefly, after gentle shaking with equal volume of PBS, the mixture was separated into two phases. The upper phase (containing stem cells, adipocytes and blood) after washing with PBS was enzymatically dissociated with 0.075% collagenase (type I)/PBS for 1 h at 37°C with gentle shaking. The dissociated tissue was then mixed with an equal volume of DMEM (GIBCO-BRL, Japan) supplemented with 10% FBS and incubated 10 min at room temperature. The solution then was separated into two phases. The lower phase was centrifuged at 1500 rpm for 5 min at 20°C. The cellular pellet was resuspended in 160 mM NH_4_Cl to eliminate erythrocytes and passed through a 40 μm mesh filter into a new tube. The cells were resuspended in an equal volume of DMEM/10% FBS and then centrifuged. Isolation resulted in obtaining 7.7 × 10^6^ of adherent cells for a primary culture from 5 g of adipose tissue (approximately; 1.0 × 10^5^ to 4.6 × 10^6^/1 g) after 7–10 days of culture. The cells were suspended in DMEM/10% FBS plated in concentration 1–5 × 10^6^ cells/75 cm^2^. The phenotype of ASCs was evaluated by flow-cytometry analysis (FC500 Beckman Coulter). The ASCs presented as a homogeneous fibroblastic cell population. Flow cytometric analysis of passage 4th cells revealed that cells were negative for CD34 and CD45, and that cells were positive for CD105 and CD90 (Data not shown).

### Differentiation of Human ASCs into Adipocytes

ASCs (passage 4–5) were plated in a 75-cm^2^ flask at a density of 1–2 × 10^4^ cells and cultured in DMEM with 10% FBS for 7 days. The medium was replaced with adipogenic medium, and the cells were cultured for 14 days.

The adipogenic media consisted of complete culture medium supplemented with DMEM-F12 high glucose, 3% (v/v) FBS, 100 nM insulin, 100 nM dexamethasone (Sigma–Aldrich, St. Louis, MO, USA), 0.5 mM isobutylmethylxanthine (Sigma–Aldrich, St. Louis, MO, USA), 60 μM indomethacin (Sigma–Aldrich, St. Louis, MO, USA), and transferrin 10 μg/ml. Media were changed every 3 days. Human ASCs were cultured in the presence of OLE from Biancolilla cv., containing 0.27 mg caffeic acid/mL and 0.37 mg oleuropein/mL, which were administered every 3 days.

### Oil Red O Staining

Staining was performed using 0.21% Oil Red O in 100% isopropanol (Sigma–Aldrich, St. Louis, MO, USA). Briefly, adipocytes were fixed in 10% formaldehyde, stained with Oil Red O for 10 min, rinsed with 60% isopropanol (Sigma–Aldrich), and the Oil Red O eluted by adding 100% isopropanol for 10 min and the optical density (OD) measured at 490 nm, for 0.5 s reading. Lipid droplets accumulation was examined by using inverted multichannel LED fluorescence microscope (Evos, Life Technologies, Grand Island, NY, USA).

### RNA Extraction and qRT-PCR

RNA was extracted by Trizol reagent (Invitrogen, Carlsbad, CA, USA; Tibullo et al., 2013). First strand cDNA was then synthesized with Applied Biosystem (Foster City, CA, USA) reverse transcription reagent. Quantitative real-time PCR was performed in Step One Fast Real-Time PCR System Applied Biosystems using the SYBR Green PCR MasterMix (Life Technologies; Malaguarnera et al., 2011; Marrazzo et al., 2011). The primer sequences used are shown in **Table 1**. The specific PCR products were detected by the fluorescence of SYBR Green, the double stranded DNA binding dye. The relative mRNA expression level was calculated by the threshold cycle (*C*_t_) value of each PCR product and normalized with that of GAPDH by using comparative 2^-ΔΔ^*^C^*^t^ method.

**Table 1:** PCR primers used in this study.

Gene	Primer forward	Primer reverse
CEBPα	TAACTCCCCCATGGAGTCGG	ATGTCGATGGACGTCTCGTG
DGAT1	CGCGGACTACAAATGGACGA	AACCAGTAAGACCACAGCCG
DLK1	TCCTCAACAAGTGCGAGACC	CTGTGGGAACGCTGCTTAGA
FABP4	AAACTGGTGGTGGAATGCGT	GCGAACTTCAGTCCAGGTCA
FAS	CGGAGGCATCAACCCAGATT	GATGGTGGTGTAGACCTTCCG
GAPDH	AGACACCATGGGGAAGGTGA	TGGAATTTGCCATGGGTGGA
IL6	CTTCTCCACAAGCGCCTTCG	CTGGCATTTGTGGTTGGGTC
IRS1	GCAACCAGAGTGCCAAAGTG	AGGTCATTTAGGTCTTCATTCTGCT
PGC1α	GGTGCAGTTTTGCCAAGGAG	TTCCTTGGGGTCCAGACAGA
PPARα	AAGAGCTTGGAGCTCGGC	TGAAAGCGTGTCCGTGATGA
PPARγ	AGAGTACGTGGGAGAAATGAC	GATGGCCACCTCTTTGCTCT
SIRT1	TGATTGGCACAGATCCTCGAA	AAGTCTACAGCAAGGCGAGC
SREBP-1c	CCCCACTTCATCAAGGCAGA	GCTGTGTTGCAGAAAGCGAA
TNF α	CTCGAGTCAGATCATCTTCTCGCACCCCG	GGAATTCTGTTCGTCCTCCTCACAGGGC
UCP-1	TGTCCTGGGAACAATCACCG	TCCAGGATCCAAGTCGCAAG

### Western Blot Analysis

Western Blot analysis was performed as previously described (Caccamo et al., 2004; Salamone et al., 2012). Primary monoclonal antibodies directed against UCP-1 (Santa Cruz, Milan, Italy), Heme oxygenase 1 (Enzo Life, Milan, Italy), pAMPK (Cell-Signaling, Milan, Italy), and β-actin (Santa Cruz, CA, USA) were used to blot the membrane (1:1000).

Protein detection was carried out using a secondary infrared fluorescent dye conjugated antibody absorbing at 800 or 700 nm. The blots were visualized using an Odyssey Infrared Imaging Scanner (Li-Cor Science Tec, Milan, Italy) and quantified by densitometric analysis performed after normalization with β-actin.

### Statistical Analyses

Statistical significance (*P* < 0.05) of differences between experimental groups was determined by the Fisher method for analysis of multiple comparisons. For comparison between treatment groups, the null hypothesis was tested by either single-factor analysis of variance (ANOVA) for multiple groups, or the unpaired *t*-test for two groups, and the data are presented as mean ± SEM.

## Results

### Partial Characterization of OLE Extracts

Olive leaves dried samples were extracted by aqueous solvent. The results show that OLE from ‘Biancolilla’ presents the highest value of both total polyphenols content and radical scavenging activity, expressed as RSA% and TEAC value, similar to that of *Nocellara* (**Table 2**). OLE from ‘Biancolilla’ was employed for the following experiment. The extract obtained by the aqueous method contains phenolic compounds that were identified and quantified by HPLC analysis and oleuropein (46.25 mg/g of dried leaves) is the most abundant antioxidant compound, followed by hydroxytyrosol glucoside and ligstroside (15 and 9.7 mg/g of dried leaves, respectively). The extract contained also luteolin-7-*O*-glucoside, verbascoside, rutin, and hydroxytyrosol, and caffeic acid, chlorogenic acid (data not shown).

**Table 2:** Total polyphenol content, radical scavenging activity, and Teac from different OLE cultivars.

Olive tree cultivar	Total Polyphenols^1^ (mg/g)	Radical Scavenging Activity (%)	TEAC^2^ (mM)
Biancolilla	40.59 ± 1.98	90.50 ± 0.56	2.82 ± 0.02
Coratina	20.17 ± 0.04	81.58 ± 0.64	2.54 ± 0.02
Nocellara	39.55 ± 2.30	90.31 ± 0.08	2.81 ± 0.00
San Benedettese	15.04 ± 0.06	76.31 ± 0.40	2.37 ± 0.01

*^1^mg of caffeic acid/g dried leaves.*

*^2^Trolox equivalent antioxidant capacity.*

### Analysis of Adipogenic Differentiation

**FIGURE 1:**
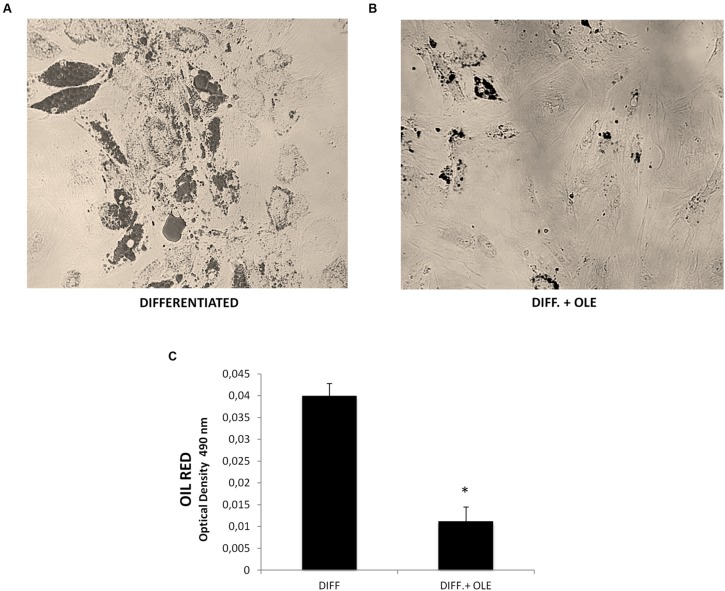
**(A,B)** Lipid droplets accumulation measured by Oil red staining in differentiated cells (14 days of adipogenic differentiation) in presence **(B)** or absence of OLE **(A)**. Adipogenesis was measured as the relative absorbance (OD 490 nm) of Oil Red at day 14 after inducing adipogenesis as described in Section “Materials and Methods” **(C)**. All values are expressed as mean ± SEM of four experiments (*n* = 4) in duplicate. (*P < 0.05 versus differentiated).

**Figure 1A** shows the positive OIL RED staining of the cells after 14 days of differentiation. Quantification of Oil Red O-stained cells showed that lipid droplets decreased following OLE treatment (**Figures 1B,C**).

**FIGURE 2:**
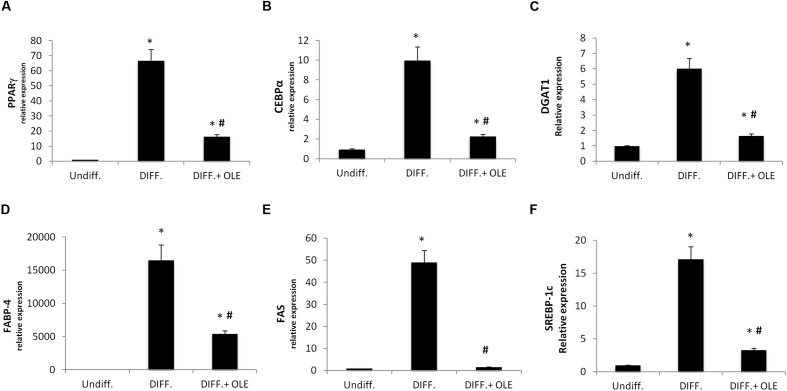
**Analysis of gene expression by Real time PCR of PPARγ **(A)**, CEBPα **(B)**, DGAT1 **(C)**, FABP4 **(D)**, FAS **(E)**, and SREBP-1c (F).** All values are expressed as mean ± SEM of four experiments (*n* = 4) in duplicate. (*P < 0.05 versus undifferentiated; ^#^*P* < 0.05 versus differentiated).

To investigate signals that might regulate the differentiation of ASCs, we analyzed the mRNA levels of PPARγ (**Figure 2A**), CCAAT/enhancer-binding protein alpha (CEBPα; **Figure 2B**), diacylglycerol *O*-acyltransferase 1 (DGAT1; **Figure 2C**), fatty acid binding protein 4 (FABP4; **Figure 2D**), fatty acid synthase (FAS; **Figure 2E**), and sterol regulatory element-binding protein 1c (SREBP-1c; **Figure 2F**).

We showed that all of these markers resulted in a significantly increase after 14 days of adipogenic differentiation.

### The Effect of Olive Leaf Extract (OLE) on the Adipogenesis

**FIGURE 3:**
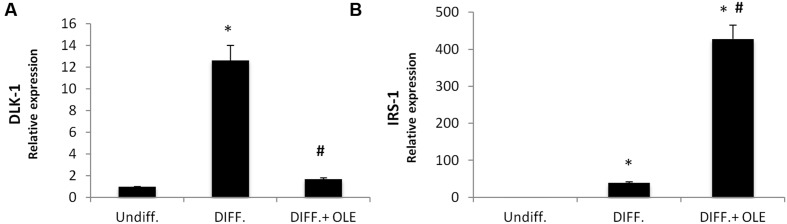
**Analysis of gene expression by Real time PCR of DLK-1 **(A)**, and IRS-1 (B).** All values are expressed as mean ± SEM of four experiments (*n* = 4) in duplicate. (*P < 0.05 versus undifferentiated; ^#^*P* < 0.05 versus differentiated).

As seen in **Figure 2**, the administration of OLE during the adipogenic differentiation was able to reduce significantly the mRNA levels of PPARγ, CEBPα, DGAT1, FABP4, FAS, and SREBP-1c (**Figures 2A–F**). Furthermore, we found a significantly reduction of Delta like 1 (DLK-1) mRNA levels by OLE (**Figure 3A**). Interesting, OLE effects show a significantly increase of insulin receptor substrate 1 (IRS-1) genes expression (**Figure 3B**).

### Analysis of Inflammatory Cytokines

**FIGURE 4:**
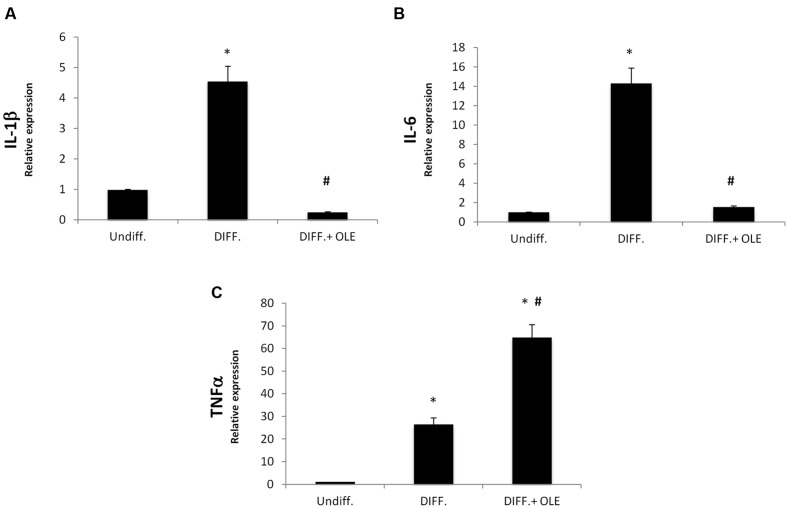
**Analysis of gene expression by Real time PCR of cytokines IL-1β **(A)**, IL-6 **(B)**, and TNFα (C).** All values are expressed as mean ± SEM of four experiments (*n* = 4) in duplicate. (*P < 0.05 versus undifferentiated; ^#^*P* < 0.05 versus differentiated).

In order to study, the eventually effect of OLE on inflammation, we investigated IL-1β, Il-6, and TNF-α expression during differentiation. We showed a significantly increase of mRNA levels of these in differentiated adipocytes (**Figures 4A–C**). The OLE treatment was able to significantly decrease the IL-1β and Il-6 mRNA levels in differentiated cells. Interesting the addition of OLE during differentiation resulted in a significant increase of TNFα gene respect to the control cells (**Figure 4C**).

### OLE Induces Thermogenic Pathway

In order to investigate the effect of OLE on the lipid metabolism, we analyzed the expression of the thermogenic pathway markers, which are characterized in brown adipocyte.

**FIGURE 5:**
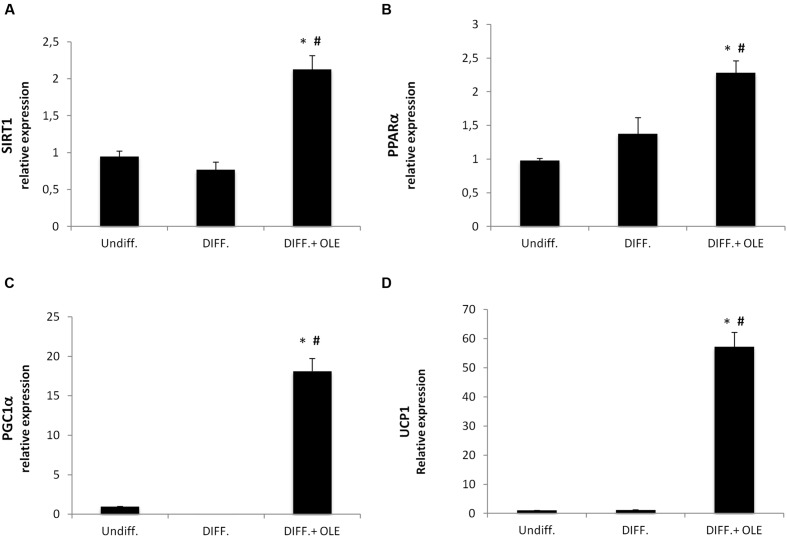
**Analysis of gene expression by Real time PCR of thermogenic pathway.** SIRT1 **(A)**, PPARα **(B)**, PGC1α **(C)**, and UCP1 **(D)**. (*P < 0.05 versus undifferentiated; ^#^*P* < 0.05 versus differentiated).

**FIGURE 6:**
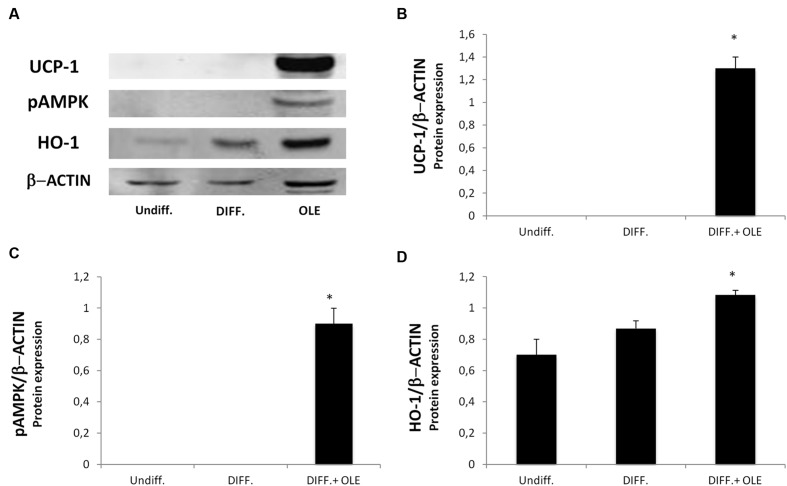
**Analysis of protein expression, evaluated by Western Blot, of cells treated with OLE during adipogenic differentiation.** The figure shows bands of western blots **(A)** and the densitometric analysis of UCP1 **(B)**, pAMPK **(C)**, and HO-1 protein expression **(D)**. All values are expressed as mean ± SEM of three experiments (*n* = 3) in duplicate. The values are expressed as ratio of protein on the β-Actin expression. (*P < 0.05 versus DIFF).

The administration of OLE during adipogenic differentiation was able to significantly increase mRNA levels of SIRT-1, PPARα and PPARγ, Pgc-1α (**Figures 5A–C**). Moreover, to study the activation of heat-generating pathway, which is the futile cycle of proton pumping through the actions of UCPs, we analyzed the expression of UCP1. OLE was able to significantly increase the expression levels of UCP1 gene and protein (**Figures 5D** and **6A,B**).

### Effect of OLE on HO-1 and pAMPK Expression

To investigate the possible role of OLE on the phosphorylation of AMP-activated protein kinase (AMPK), we performed a western blot analysis. The OLE treatment resulted in a significantly increase of pAMPK expression during adipose differentiation (**Figures 6A,C**). HO-1 gene and protein expression, an antioxidant protein, resulted in an significantly increase in OLE treated cell cultures after 14 days of adipogenic differentiation respect the differentiated untreated cells (**Figures 6A,D**).

## Discussion

Several studies suggest that flavonoids content in OLE may account for the overall radical scavenging activity potential. This latter effect is dependent on the levels of each single phenolic compound and other leaf constituents in relation to phenological stage (Papoti and Tsimidou, 2009). The leaf represents a source of antioxidants for the high total polar phenol content and antiradical potency, which is also related to the cultivar considered. This work represents the first application of an aqueous OLE from Sicilian cultivar.

In the present study, we showed that OLE treatment during adipogenic differentiation of adipose derived human mesenchymal stem cell induces thermogenesis in adipocytes by activation of uncoupling protein UCP1, SIRT-1, PPARα, and Pgc-1α. UCP1 is expressed in both brown and brown-like adipocytes, which are also known as recruitable beige or brite cells. Furthermore, UCP1 in brown-like adipocytes induces thermogenesis by activation of mitochondrial futile cycle (Poher et al., 2015).

It was demonstrated that SIRT1, an NAD^+^ dependent type III deacetylase sirtuin, potentiates BAT function enhancing glucose tolerance (Boutant et al., 2015) and inducing resistance to obesity (Wang et al., 2013). SIRT1 exerts its function by interacting with PPARα followed PGC-1α activation (Purushotham et al., 2009). PPARα in human WAT induces brown fat gene expression such as UCP1 and PGC-1α (Mandard et al., 2004; Hondares et al., 2011) conferring to it a role of distinctive marker of BAT respect to the WAT phenotype (Villarroya et al., 2007). In addition, Pgc-1α expression is essential for thermogenic activation of brown adipocytes (Uldry et al., 2006) and thermogenic genes in WAT (Kleiner et al., 2012). Our data suggest that OLE treatment resulted in a decrease of PPAR gamma, FABP4, FAS, DGAT1 (Harris et al., 2011), and SREBP-1c gene expression during the differentiation, confirming that its administration is able to reduce fatty acid accumulation. Moreover, decrease in IL-1β and IL-6 expression in the adipocyte differentiated whit OLE respect to the normal adipocyte confirms a reduction of inflammation. Interestingly, we found an overexpression of TNFα by OLE. However, this unexpected data is consistent with another study demonstrating that TNFα signaling, in the presence of adipogenic induction cocktail, may activate β-catenin/TCF target gene expression thus suppressing CEBPα expression and inhibiting adipogenesis (Cawthorn et al., 2007). Furthermore, a previously report showed that treatment with an antioxidant may increase TNF-α *via* stabilization of its mRNA (Nathens et al., 2001). In our study, we found that OLE treatment during differentiation led to an overexpression of heme oxygenase 1 (HO-1), an inducible enzyme known for its protective and antioxidant effect in vascular disease and in the metabolic syndrome (Novo et al., 2011; Barbagallo et al., 2012, 2014; Marrazzo et al., 2014). In addition, our group previously suggested that the induction of HO-1 decreases adipogenesis by its antioxidant effect (Barbagallo et al., 2010; Burgess et al., 2012). Furthermore, in adipocyte treated with OLE we found an increase of IRS1 gene expression, which have been previously reported to play an important role in brown adipocyte differentiation. To this regard, a previous study demonstrated a defect in differentiation of cells lacking IRS-1, which could be restored by IRS-1 gene activation (Tseng et al., 2004). Moreover, the decrease of DLK1 gene expression by OLE is consistent with other study showing that DLK-1 expression inhibits heat production in BAT (Rakhshandehroo et al., 2012).

In order to elucidate the molecular mechanism by which OLE modulates the adipogenesis, we measured the protein level of phosphorylated AMPK, which is known to be one among multiple players in metabolic switch. Treatment with OLE induced the phosphorylation of AMPK. Activation of AMPK has emerged as an important target for the treatment of metabolic syndrome since its activation is necessary for the inhibition of adipogenesis in 3T3-L1 cells by phytochemicals (Kim and Kong, 2010; Ruderman et al., 2013). One possible main limitation of present study is that it was conducted only *in vitro* and under non-pathological culturing condition; furthermore, our work is not investigating so far the measurement of basal mitochondrial metabolism.

## Conclusion

Our data suggest that OLE significantly decreases the expression of molecules involved in adipogenesis and upregulates the expression of molecules involved in thermogenesis modulating lipid metabolism. Taken together, our results suggest that OLE may promote the brown remodeling of WAT inducing thermogenesis and improving metabolic homeostasis.

## Author Contributions

RP, GL, and IB conceived and coordinated the study and wrote the paper. RP, JM, GS, CR, MR, LV, GV, and IB designed, performed, and analyzed the experiments. MR provided technical assistance and contributed to the preparation of the figures. All authors reviewed the results and approved the final version of the manuscript.

## Conflict of Interest Statement

The authors declare that the research was conducted in the absence of any commercial or financial relationships that could be construed as a potential conflict of interest.
